# Granulomatous skin lesions complicating Varicella infection in a patient with Rothmund-Thomson syndrome and immune deficiency: case report

**DOI:** 10.1186/1750-1172-5-37

**Published:** 2010-12-08

**Authors:** Lien De Somer, Carine Wouters, Marie-Anne Morren, Rita De Vos, Joost Van Den Oord, Koenraad Devriendt, Isabelle Meyts

**Affiliations:** 1 Department of Paediatrics, University Hospitals Leuven, Leuven, Belgium; 2 Department of Pediatric Rheumatology, University Hospitals Leuven, Leuven, Belgium; 3 Department of Dermatology, University Hospitals Leuven, Leuven, Belgium; 4 Department of Morphology and Molecular Pathology, University Hospitals Leuven, Leuven, Belgium; 5 Center for Human Genetics, University Hospitals Leuven, Leuven, Belgium; 6 Department of Pediatric Immunology, University Hospitals Leuven, Leuven, Belgium

## Abstract

Rothmund-Thomson syndrome (RTS)(OMIM 268400) is a rare autosomal recessive genodermatosis characterized by poikiloderma, small stature, skeletal and dental abnormalities, cataract and an increased risk of cancer. It is caused by mutations in RECQL4 at 8q24. Immune deficiency is not described as a classical feature of the disease. Here we report the appearance of granulomatous skin lesions complicating primary Varicella Zoster Virus infection in a toddler with Rothmund Thomson syndrome and immune deficiency. Although granulomatous disorders are sometimes seen after *Herpes zoster*, they are even more rare after Varicella primary infection. Granulomas have hitherto not been described in Rothmund-Thomson syndrome. With this report we aim to stress the importance of screening for immune deficiency in patients with Rothmund-Thomson syndrome.

## Background

Rothmund-Thomson syndrome ((RTS) OMIM 268400) is a rare autosomal recessively inherited genodermatosis with a heterogeneous clinical presentation. It is characterized by a characteristic facial rash appearing in infancy (poikiloderma), short stature, radial ray defects, variable degrees of osteopenia, sparse scalp hair, eyelashes and eyebrows, dental abnormalities and cataract. Moreover, RTS patients are at increased risk of cancer (especially osteosarcoma and non melanoma skin cancer, but also leukemia and a range of other tumors). The syndrome was originally described in 1868 by the German ophthalmologist Rothmund in patients with rapidly progressive juvenile cataract associated with skin abnormalities [1]. In the first half of the 20^th ^century the English dermatologist Thomson mentioned two patients with cutaneous lesions that were similar to those reported by Rothmund, but without any ocular lesions [2]. In 1953 these two medical conditions were designated as the Rothmund-Thomson syndrome [3].

Following the association of RTS to mutations in RECQL4 at 8q24, two clinical-molecular subsets of RTS have been proposed [4,5]. Mutations in RECQL4 are present in approximately one third of the clinically diagnosed RTS patients (RTS type II) and these patients phenotype is characterised by poikiloderma, bone defects and an incresased risk of osteosarcoma. In RTS type I patients, no RECQL4 mutations are found and poikiloderma, juvenile cataracts and ectodermal dysplasia dominate the clinical presentation [4]. The human RECQL4 gene consists of 21 exons spanning 6.5kb and encodes for a 1208 aminoacids protein [6]. There is evidence that RECQL4 plays multiple key roles in DNA metabolism, as it is involved in single-stranded DNA annealing activity, DNA replication, double strand break repair, and repair of UV or ionizing radiation induced DNA damage [7]. RTS and the function of RECQL4 have recently been excellently reviewed by Larizza et al [8].

We present a patient with RTS and immune deficiency who developed granulomatous skin lesions after primary *Varicella zoster virus *(VZV) infection. To our knowledge granulomatous skin lesions have not been reported in RTS. Although granuloma formation has been described in Herpes zoster, it is rare after primary VZV infection.

## Case presentation

**Figure 1:**
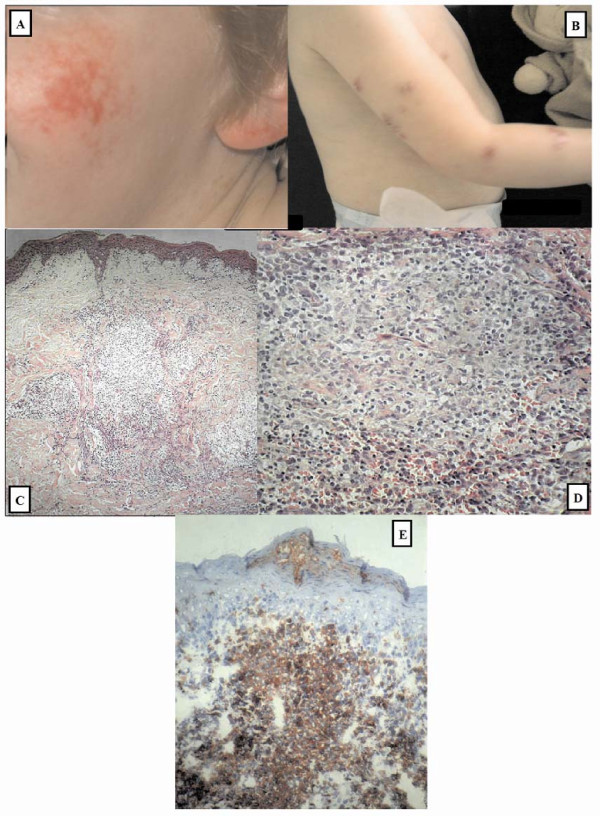
**Clinical and histological view of granulomatous lesions**. A. Typical poikiloderma lesions on the cheek and ear. B. Deeply red-violet granulomatous skin lesions on arms. C. Low power microscopy of the skin lesion reveals superficial and deep inflammation without repercussion on the epidermis, and several lightly stained granulomas, surrounded by inflammatory cells. Haematoxylin and eosin stain, original magnification × 10. D. High power of a granuloma reveals a mixture of epithelioid macrophages, scattered lymphocytes and lack of caseous necrosis; the granuloma is surrounded by a narrow rim of mononuclear inflammatory cells. Haematoxylin and eosin stain, original magnification × 25. E. Immunohistochemical staining highlighted the granulomas, composed of CD68(+) macrophages. Immunohistochemical staining for CD68, original magnification × 10.

The propositus is the second child of non-consanguineous healthy parents of Kaukasian descent. She was born term with weight, length and head circumference below the third percentile. At the age of 6 months she developed serpiginous erythematous lesions on the facial skin (Figure 1A). At the age of 18 months, she was diagnosed with chickenpox. Four weeks after appearance of the first VZV lesions, the spots changed into deeply red-violet indurated and painful skin lesions on the cheeks, the ears and arms (Figure 1B). Talc powder had not been used topically. The course of the chicken pox infection was otherwise uncomplicated. Furthermore, there was a history of recurrent pyogenic upper and lower respiratory tract infections with chronic purulent rhinorrhea and chronic productive coughing.

Upon physical examination at the age of 2y, we saw a girl with weight and stature < P3. Inspection of the skin showed atrophic and hyperpigmented skin lesions, mixed with café-au-lait lesions. Also, the girl had a pointed nose as well as long, fine fingers with aberrant, finger-like thumbs with diminished flexion. There was clubbing on inspection and crackles on lung auscultation. Heart tones were normal as were peripheral arterial pulsations. Inspection of ears, nose and throat showed bilateral glue ears, purulent rhinitis. Inspection of the oral cavity showed that the lateral incisors were joined with the mandibular canine teeth. Clinical neurological examination showed no abnormalities. Ophthalmologic examination was normal. Full immunological investigation at the age of 3y revealed normal CD4(+) counts (1572/mcl (normal range 500-2400), low CD8(+) (191/mcL (normal range 300-1600/mcL)) and CD16/56(+) (12/mcL (90-900/mcl)) cells. Naive T cells (CD4(+)CD45RA(+)CD27(-)) were 72% of total CD4(+) T cells (normal range 65-95%). Lymphocyte proliferative responses to Candida, Tetanus toxoid, VZV, PMA/ionomycin, PHA, ConA were all normal. Total CD19(+) cells were normal (929/mcl (normal range 200-2150)) but percentage of switched memory B cells was low (CD19(+)CD27(+)IgM(-)IgD(-)) (1% (normal range for age 1.5-4.1%). The values of CD8 cells, NK cells and class-switched B cells remained low at ages 4.5 and 6y. Also, IgG2 (0.47 g/L (normal range 0.63-3.6 g/L)) and IgM (0.27 g/L (normal range 0.34-1.34 g/L) were low. Specific antibodies against *Poliovirus*, measles and *Hepatitis B virus *were lacking despite adequate immunization. VZV IgG was present. Iso-hemagglutinin titers were measured twice and were low, both at the age of 3 years (1/4) as well as at the age of 6 years (1/1). Moreover anti-pneumococcal antibody response was low. These findings point to a defect in anti-polysaccharide antibody responses. Computerized tomography of the lungs revealed various sites of consolidation, developing bronchiectasis and mosaic perfusion. Histological examination of an indurated skin lesion on the arm revealed superficial and deep non-caseating granulomas (Figure 1C-E). Histochemical stains for fungi and mycobacteria (Ziehl-Neelsen) were negative. Electron microscopy examination was performed and showed no evidence of foreign bodies or presence of micro-organisms as the cause of granuloma formation. Immunohistochemistry did not show evidence of immune complex- or complement-mediated pathology. VZV could not be detected in the granulomata by polymerase chain reaction. Tissue cultures for bacteria, mycobacteria or fungi were negative. No 16sPCR, pan-mycobacterial or *Mycobacterium tubercolusis *PCR was performed.

A presumptive diagnosis of Rothmund-Thomson syndrome was made and supported by the finding of a delayed bone age (2 y 10 m 24 d versus civil age of 3 y 11 m 18d) and of metaphyseal translucencies in tibia and ulna. Genetic analysis confirmed the diagnosis as the girl was heterozygous for 2 mutations in the RECQL4 gene (1048-1049delAG; 1391-1G > A).

The patient is now 6 years old and doing well under prophylactic antibiotics (amoxicilline) and subcutaneous immunoglobulin therapy, daily chest physiotherapy and nose washes, the combination of which significantly reduced the morbidity of recurrent respiratory infections. She picked up weight and is now thriving at P3. The granulomatous skin lesions gradually diminished and disappeared completely over a time span of several months.

## Discussion

We describe a patient with Rothmund-Thomson syndrome and humoral immune deficiency with granulomatous skin lesions after a VZV infection. Up till now, granulomatous skin lesions have not been reported in patients with RTS.

Granulomas are formed as a result of exaggerated cellular immune response to one or more external or self-antigens and a subsequent accumulation of activated macrophages and CD4(+ T cells at an inflammatory site. Several organs can be affected: mainly the lungs, but also the skin, the liver, lymphoid tissue and the spleen. Granulomas can be seen in the context of primary immunodeficiency disorders. Up to 20% of patients with common variable immunodeficiency (CVID) develop cutaneous and systemic granulomatous inflammation [9,10]. Cutaneous and systemic granulomas have also been observed in patients with cartilage hair hypoplasia (unpublished observation), ataxia-telangiectasia, etc. Obviously, granulomatous inflammation is the hallmark of chronic granulomatous disease. Our patient had an increased frequency of respiratory infections associated with immune deficiency defined by low IgG2 and a diminished specific antibody response together with low CD8(+)T cells and low NK CD16(+) CD56(+)cells. Moreover she developed granulomatous skin lesions after primary VZV infection. The temporal and spatial association between the skin lesions and the VZV infection is not a proof of VZV as the cause of the granuloma formation. However, the appearance of granulomas does point to the possibility of immune deficiency or immune dysregulation. In CVID patients, granulomas are believed to develop in response to intracellular pathogens [11,12]. Also, there seems to be an association with lymphopenia, an inverted CD4/CD8 ratio and decreased T-cell proliferation in response to mitogens and antigens[10]. Wehr et al. found that in CVID granulomatous inflammation is associated with reduced numbers of memory B cells[13]. Our patient presented with antibody deficiency and low CD8 counts as well as a low percentage of switched memory B cells.

Originally, immunodeficiency was not believed to be a characteristic feature of RTS. Recently, an increasing number of reports on increased susceptibility to infections and immune deficiency in RTS are emerging. Kubota reported an RTS patient with susceptibility to sinopulmonary infections and IgG4 deficiency [14]. Ito reported a 4-year-old Japanese boy with RTS presenting with herpes encephalitis at 5 months of age. The serum level of immunoglobulin G was low and the responsiveness of peripheral blood mononuclear cells to bacterial superantigens was poor [15]. Broom described a patient with molecularly confirmed RTS who at the age of 7 months was admitted with *Pneumocystis jiroveci *pneumonia. Immunophenotyping revealed a T^-^B^+^NK^- ^phenotype with agammaglobulinemia consistent with severe combined immunodeficiency. The patient received an umbilical cord blood transplant with complete immune reconstitution [16]. In 2007 Reix et al described 2 pediatric patients in whom localized bronchiectasis developed [17]. Thus, certainly a subset of patients with RTS seems to suffer from clinically relevant immune deficiency. Due to the limited number of reports we can only speculate on a genotype-phenotype correlation in this syndrome with respect to the immune deficiency. Most patients reported with significant immune deficiency did have RECQL4 mutations. The pathophysiology of immune deficiency in RTS with RECQL4 mutations can probably be seen in the context of the multiple roles of RECQL4 in DNA metabolism, as summarized above. Thus, RTS II shares similarities with chromosomal breakage syndromes like ataxia telangiectasia, nijmegen breakage syndrome in which immunodeficiency is often present, as well as an increased risk for cancer. Moreover, RECQL4 is highly expressed in the thymus and KO mice have smaller thymi, suggesting a role for RECQL4 in T cell development [18]. The presence of immune deficiency or aberrant immunological function is in agreement with the emergence of granulomas in our patient. In patients recently affected by *Herpes zoster *several cutaneous reactions have been described with granuloma annulare and other granulomatous disorders being most common. Foreign body granulomas secondary to the use of talc have been described; this possibility was excluded in our patient [19]. Most patients proved to be immunosuppressed for various reasons (age, hematological malignancy, HIV, solid tumors), others showed no apparent immune deficiency [20,21]. VZV DNA is inconsistently demonstrated in the lesions, whether or not they manifest early (weeks) or late (months) after the *Herpes zoster *infection [22]. Nikkels et al. suggest that VZV envelope glycoproteins may trigger granuloma formation [23].

The occurrence of a new skin disorder exactly at the site of another one, already healed was recognized as a distinct dermatologic phenomenon named "istotopic response". Two cases of isotopic response at the site of VZV infection were described in the extended literature review in this paper [24]. However, we are aware that we have not rigorously excluded a potential atypical mycobacterial infection.

## Conclusion

Immune deficiency or immune dysregulation is an increasingly reported characteristic of Rothmund-Thomson syndrome. A high index of suspicion of immune deficiency is warranted in patients with RTS as appropriate therapeutic measures may have significant impact on short- and long-term morbidity and mortality. Immune deficiency and/or immune dysregulation predispose to aberrant responses to infection or noxious stimuli in the form of granulomatous inflammation. To the best of our knowledge, this is the first report of granulomas in a patient with Rothmund-Thomson syndrome. The immunodeficiency in our patient may have contributed to the development of granulomas. In our opinion, screening for immune deficiency is warranted in every patient with Rothmund-Thomson syndrome and more so in the event of recurrent infections or if an unusual course of a common infection is encountered.

## List of abbreviations

CVID: Common Variable Immune Deficiency; RTS: Rothmund-Thomson syndrome; VZV: *Varicella Zoster Virus*.

## Competing interests

The authors declare that they have no competing interests.

## Authors' contributions

LDS drafted the manuscript and participated in clinical care for the patient. CW, MM aided in preparing the manuscript and participated in clinical care. RDV and JVDO aided in preparing the manuscript and aided in the granuloma work-up. KD aided in genetic diagnostics and in preparing the manuscript. IM drafted and finalized the manuscript, characterized the immune deficiency and is coordinating clinical care for the patient. Each author has critically revised the final version of the manuscript and all authors have read and approved the final manuscript.

## Consent

Written informed consent was obtained from the patient's parents for publication of this case report and any accompanying images. A copy of the written informed consent is available for review by the Editor-in-Chief of this Journal.
